# Field Phenomics: Will It Enable Crop Improvement?

**DOI:** 10.34133/2021/9871989

**Published:** 2021-09-02

**Authors:** David M. Deery, Hamlyn G. Jones

**Affiliations:** ^1^CSIRO Agriculture and Food, Canberra, ACT, Australia; ^2^Division of Plant Sciences, University of Dundee, UK; ^3^School of Agriculture and Environment, University of Western Australia, Australia

## Abstract

Field phenomics has been identified as a promising enabling technology to assist plant breeders with the development of improved cultivars for farmers. Yet, despite much investment, there are few examples demonstrating the application of phenomics within a plant breeding program. We review recent progress in field phenomics and highlight the importance of targeting breeders' needs, rather than perceived technology needs, through developing and enhancing partnerships between phenomics researchers and plant breeders.

## 1. Introduction

The continued breeding of high-yielding and stress-tolerant varieties is one of the key challenges facing agriculture in the coming years. Although the development of genomic selection methods [1, 2] has greatly aided the breeding process, there is still a widely recognized gap in the development and application of tools for equally rapid phenotyping of the resulting germplasm [3–6]. For many years, plant breeders and researchers have been interested in enhanced methods to select lines with specific physiological and agronomic characters that contribute to stress tolerance and to final yield, but the traditional measurements for quantitative studies have tended to be labor-intensive and not readily capable of scaling up to allow screening of large numbers of distinct lines, especially in the field. Conventional phenotyping, the visual assessment of plant traits, is a mainstay of plant breeding but widely accepted as subjective and laborious (refer to discussion in [7]).

Early in the 2000s with the rapid development of genotyping technology, the general term of phenomics was widely adopted to cover automated plant screening work (although the terms phenotype and genotype originated over a century ago [8]). In the past decade, the development of robotics, advanced imaging, and data analysis technologies opened up new avenues for high-throughput screening, but the cost of the necessary facilities led to the establishment of a number of specialised centres worldwide, of which the Australian Plant Phenomics Facility (with its nodes in Canberra and Adelaide) was one of the first. In addition, initiatives to create communities of practice and further phenomics have been established including various national and international networks, including this journal, Plant Phenomics [9, 10]. There is substantial international effort in the development of plant phenotyping, with many regional- or national-level activities such as the UK National Plant Phenotyping Network, the German Plant Phenotyping Network, the European Plant Phenotyping Network (now replaced by EMPHASIS), and the North American Plant Phenotyping Network. The International Plant Phenotyping Network (IPPN), which acts as a forum for phenotyping activity, recently conducted a survey of members that highlighted “field phenotyping” as the top priority in phenotyping, followed by “Abiotic stress” and “Data management” (https://www.plant-phenotyping.org/ippn-survey_2016). Emphasis in the development of field phenotyping is particularly important both because it is critical for the deployment into plant breeding, but also because it is still much more challenging than laboratory or glasshouse phenotyping and less advanced.

In recent years, many review publications (e.g., [6, 7, 11, 12]) have highlighted the importance of phenomics as an enabling tool for crop improvement through research and plant breeding and many encouraging results have been reported (discussed below). It is timely, therefore, to review the prospects for the application of phenomics as an enabling tool for crop improvement through research and plant breeding. Because phenomics is such a large field and with many researchers actively involved, the review is limited rather specifically to imaging approaches, aboveground phenotyping, and especially their application to phenotyping for cereals.

## 2. The Challenge of Augmenting Conventional Plant Breeding

**Figure 1:**
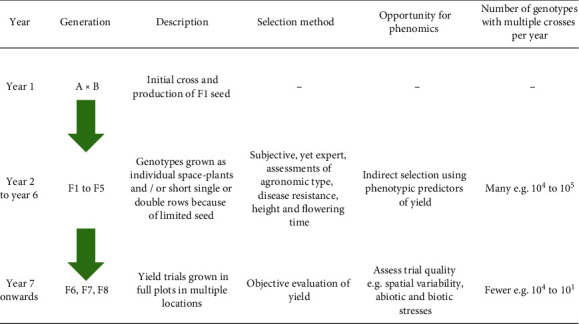
Elementary summary of the various stages of cultivar development within a plant breeding program for a self-pollinating species. The description of planting formats used in the early generations are indicative in the sense that various breeding strategies exist [27, 28], within which the planting format will differ. The opportunity for phenomics through indirect selection is particularly strong in the early stages of the breeding program, when plants are grown in a space-plant configuration and reliable yield estimates are not attainable; assessment during these stages is largely subjective. For the purpose of discussion and to highlight the scale of assessment required, the range in the number of genotypes under assessment is indicated.

Conventional plant breeding, in both the public and private sectors, has successfully contributed to increased yields in the world's major crops over the past century [13]. However, there is evidence that relative yield progress through breeding has fallen as yields have increased (to less than 1% per year in wheat [13–18]). In order to maintain and improve rates of breeding progress, transdisciplinary approaches have been proposed to augment the process of plant breeding [19]. These include, among other approaches, the use of genomic selection and crop simulation modelling in conjunction with germplasm evaluation in carefully designed managed environments (MEs) representative of the target population of environments (TPEs) [20–23]. A universal concept for evaluating performance of a given breeding strategy is the genetic gain, ∆*G*, for a particular target trait (e.g., grain yield), colloquially known as the “breeder's equation,” which describes the response to selection per selection cycle, *c* [24].

(1) $$\begin{array}{c} \Delta G=\frac{h^{2}\sigma_{p}i}{c}, \end{array}$$

with *h*^2^ the narrow-sense heritability, *σ*_*p*_ the phenotypic standard deviation of the target trait, and *i* the selection intensity. Arguably for breeders to adopt phenomics technology, it would need to result in improved ∆*G* and/or provide an economic benefit (e.g., through reducing labor). Others have identified the opportunity for phenomics to positively influence the parameters in Equation (1) through indirect selection for yield in the early generations of a breeding program [7, 12, 25]. This opportunity is highlighted in Figure 1. In the case of a self-pollinating species like wheat, selection for yield in the early generations of a breeding program is essentially subjective, relying on the expert skill of the breeder (yet this approach is highly effective [13]). It is not until after several generations that for a given genotype, there is sufficient seed and homozygosity to reliably estimate grain yield on a plot of sufficient size with bordered rows, so that confounding factors due to edge effects can be mitigated [26]. This is shown in Figure 1, together with an indication of the range of planting formats used at the different stages. The planting formats in the early stages of a breeding program may comprise single plants, short-single, or short-double rows, collectively defined herein as space-plant configurations. Finally, for the purpose of discussion to highlight the scale of assessment required, the range in the number of genotypes under assessment at the various stages is indicated [27].

Breeders have at their disposal multiple selection strategies [27–29], and several of these were recently summarised within the context of indirect selection for yield in early-generation plantings of small grained cereals [25]. These authors [25] argued that these methods, although varied, comprise at least a single generation where the material is grown in a space-plant configuration, prior to evaluation in yield plots. And that therefore, opportunities exist to improve breeding efficiency via indirect selection for yield among material grown in a space-plant configuration. Additionally, a perhaps less conventional method utilises so-called “honeycomb selection designs” where phenotyping for selection occurs on individual plants grown at low density (100 cm plant spacing) without competitive interactions to maximise phenotypic expression and variation (see [30] and references cited therein). Thus, an opportunity for the phenotyping community lies in the potential development of methods targeted at space-plant configurations.

In the case of breeding for potential yield in small-grain cereals, a range of candidate traits for indirect selection for yield in early generations were recently reviewed [25]; stomatal conductance, fruiting efficiency (defined as the ratio of the number of grains per spike to the dry weight of the spike at anthesis [15]), and harvest index were identified as the most promising. For these three traits, the review [25] identified experimental evidence establishing that phenotypic variation expressed in a space-planting, typical of breeder's early-generation nurseries, was reliably expressed in a full plot with bordered rows to mitigate competition edge effects [26]. In addition, a range of examples were cited linking these traits to increased yield potential attained through conventional plant breeding. One such example is the concomitant increase in stomatal conductance associated with yield potential in many C3 species [31].

The potential success of early-generation selection in improving genetic gain per unit of cost is largely dependent on the development of reliable and cost-effective phenotyping methods that are scalable across the many thousands of individuals in a particular breeding program (Figure 1). Yet, despite encouraging results, the application of phenotyping within plant breeding is, arguably, still in its infancy [30, 32]. The latter is potentially hindered by the observation that a significant proportion of contemporary phenotyping studies are targeted at large plots where a reliable measure of grain yield is also attainable. Such phenotyping applied to large plots is of limited utility for breeding, as breeders frequently measure grain yield on large plots as an objective estimate of performance. As discussed elsewhere [7, 12] of potentially far greater benefit to breeding is the capacity for phenomics to routinely screen early-generation material grown in space-plant configurations where reliable yield estimates are not possible owing to edge effects [26].

Indeed, few authors have discussed the implementation of phenomics at the scale required to support a breeding program (two notable exceptions include [22, 33]). Furthermore, although the research community may be optimistic about the role of phenomics in crop improvement, it is noted that phenomics, in many cases but not all, is often based on fundamental crop physiology and there are only a handful of studies successfully reporting the application of physiological-based selection on crop improvement (refer to examples in [25]) (indeed, the contribution of crop physiology to crop improvement has been questioned in the past [34]). There are even fewer examples of the use of phenomics in conjunction with indirect selection in a breeding program (granted, commercial breeding programs may not publish their results in order to maintain competitive advantage). Nevertheless, promising results from indirect selection using phenomics predictors in experimental systems, as opposed to actual breeding programs, have been reported (e.g., [35, 36]) and are discussed in more detail below (Section 6). This review is mainly aimed at assessing what is required in order to extend the application of phenomics to actual breeding programs. In order to address this question, we must firstly review the recent technical advances in phenomics.

## 3. Sensor Carrier Systems

High-throughput phenotyping technologies are being developed both for the laboratory and controlled environment as well as for field studies: we concentrate here on the latter, though will consider in passing some aspects of greenhouse systems (e.g., conveyor belt systems) where imaging is used. Field studies are generally more relevant for potential application within a breeding program. The traditional field approach, conventional phenotyping, involves walking the plots and recording characters such as height, disease susceptibility, flowering time, and agronomic type (e.g., awns and ear structure); however, this approach is slow and labor-intensive so the search has been on for ways in which to speed up such screening activities using imaging. In the following, consideration is also given to the time-dependency of particular measurements with instantaneous snapshots of the whole trial preferable for weather dependent variables, such as canopy temperature, but slower methods (e.g., mobile “buggies”) are suitable for slowly changing variables (e.g., growth-related variables).

### 3.1. Fixed Sensor Points with Greater Field of View (Masts, Balloons, and Cherry Pickers)

An early approach to canopy imaging was to raise the height of the imagers either by mounting cameras on high stands/masts or cherry pickers or even on Helium balloons held above the crop [37]. A potential advantage of Helium balloons is that they can be tethered up to 100 m or so above the crop, giving a correspondingly large field of view, while the use of poles, stands, or cherry pickers is practically limited to a height of about 15 m, and at least for cherry pickers are expensive to hire or use. Although it is possible to enhance the area of canopy viewed by using an oblique view angle [37, 38], this introduces additional complications relating to varying apparent size of plots and varying distances with the need for more sophisticated plot extraction algorithms [39, 40]. Although these approaches to elevation of the camera have some potential advantages, in practice, the limitations of the lower-level mounts and the susceptibility of the higher systems (especially balloon-mounted systems, but even cherry pickers) to wind mean that they are unlikely to be the method of choice in the future, except in the case of small experiments. As an alternative to imaging, Wireless Sensor Networks (WSNs) comprising many fixed inexpensive individual sensors (e.g., like the ArduCrop, for canopy temperature) can provide extremely useful information, especially where continuous records are needed for physiological studies [41–43], but are unlikely to have the coverage of a buggy or aerial imaging approaches for high-throughput phenotyping applications, as discussed below. Alternatively, fixed sensors may enable improved characterisation of target growing environments, aka “envirotyping,” and thereby improved understanding of genotype-by-environment interactions [44, 45].

### 3.2. Field “Buggies”

Another approach to achieving high precision imaging of canopies is the use of mobile field “buggies” [46–48] equipped with cameras and laser scanners to measure crop height and potentially even canopy structure. Other sensors such as fluorescence imagers, hyperspectral imagers, and thermal imagers can also be mounted on such platforms. Field buggies can typically only move at a maximum of about 6 to 8 km h^−1^ and sometimes much less. For a typical 2 m swath at 5 km h^−1^, this would equate to about 1 hectare in an hour. Over this period, canopy temperature or photosynthesis will vary substantially. Therefore, buggies are well suited for characters that change only slowly (over at most a daily time scale, e.g., nitrogen status, leaf growth, or canopy height) but are likely to be much less useful for characters such as photosynthesis or canopy temperature that change dynamically with environmental conditions, as methods are needed to normalise the data to account for time differences in sampling [37, 49, 50]. For example, normalising the canopy temperature data “per pass of data collection” increased the average repeatability from the Phenocart system from 0.34 to 0.55 [50].

The use of such mobile systems, whether the earlier Phenomobile [46] or the Phenomobile Lite [48] (which is much smaller and lighter), the INRA Phenomobile [51], the “Field4Cycle” (developed by Forschungszentrum Jülich [52]) or “PhenoTrac 4” [53, 54], or even the mounting or towing (e.g., “BreedVision” system [55]) of appropriate sensors on tractors during standard crop management [33, 56, 57], can readily replace and enhance a wide range of manual and other observations that are currently frequently required in plant breeding. For example, measurements such as height, Normalised Difference Vegetation Index (NDVI) as a measure of canopy cover, and LiDAR measurements may give useful information on canopy structure and even lead to indirect estimates of aboveground biomass and even final yield, though they are not as accurate as gravimetric measurements [48, 58]. Presently, indirect estimates from imagery are subject to significant error and so only give useful relative data for similar varieties in any one trial. There is much interest in making autonomous buggies that can operate continuously without human intervention (e.g., [59]), though they have some way to go before they can replace manned buggies. A number of companies have developed track-mounted field phenotyping facilities or platforms such as the “Field Scanalyzer” [60], which is capable of scanning an area 115 m by 11 m, or the “PhenoField” [61] which comprises eight moveable rain-out shelters and precision irrigation to impose varying levels of drought (total area of 0.5 ha). These tend to be much less flexible in use than buggies, as they usually involve quite heavy engineering and fixed tracks, thus greatly limiting their flexibility and the area of crop that can be studied, so are likely to be of less use for high-throughput screening at the scale required within a breeding program (Figure 1), though they may be valuable for developing appropriate screening protocols.

From a practical standpoint, the importance of measuring many individual genotypes in a short time period was recently highlighted by demonstrating that travelling at right angles to the direction of sowing and simultaneously scanning multiple plots minimised the travel distance considerably when compared to travelling over each plot in the direction of sowing [33]. While it was acknowledged that travelling over each individual plot may improve the data quality, the trade-off for slightly reduced data quality and higher throughput was highlighted as a particular advantage for a plant breeding program.

### 3.3. Airborne (Including Unpiloted and Piloted Aircraft)

The most rapid and probably the most promising approaches to field phenotyping involve the use of airborne sensor platforms, whether these are piloted light aircraft or helicopters or unmanned aerial vehicles (UAVs also known as Remotely Piloted Aircraft Systems (RPASs) or simply “drones”) [62]. The use of airborne sensor platforms allows much more rapid collection of images than is possible from buggies on the ground. The more rapid ground coverage by airborne systems (especially fixed wing UAVs and piloted aircraft) reduces the time required to cover experiments, potentially enabling the effective study of more rapidly varying characters such as photosynthesis or transpiration. Although UAVs in Australia are limited to an altitude of 120 m thus limiting their coverage, this limitation can be overcome with piloted aircraft or helicopters [43, 63, 64].

The increasing availability of low cost UAVs that allow preprogrammed flight paths for rapid imaging of whole experiments, when combined with appropriate software for image mosaicking (e.g., Agisoft (http://www.agisoft.com), Pix4D (https://www.pix4d.com), Blue Marble Geographics Global Mapper (http://www.bluemarblegeo.com), and Autostitch [65]), makes a strong case for their wider use. For useful reviews of the commercial softwares and algorithms available for accurate automated or semiautomated image mosaicking, see Zhao et al. [66] and Gómez-Candón et al. [67]. However, limitations include flying/licensing restrictions, and in Australia, these include the need for a licence for commercial work using UAVs larger than 2 kg (UAVs that weigh less than 2 kg are exempt) (https://www.casa.gov.au). These lighter devices have only a rather limited payload and equally limited range, making them more suitable for smaller experiments using conventional RGB cameras.

The choice of platform ultimately depends on a range of features such as the type of characters being evaluated (e.g., whether they are rapidly or slowly changing, or whether they require high resolution or where low resolution is adequate for average values) (for reviews on this subject, see [68, 69]). Similarly, the choice of platform also depends on the payload of cameras, flexibility needed, and costs. There are important trade-offs between speed of flight, flight altitude, and spatial resolution; guidance on optimisation of these properties for any particular situation can be provided by software such as the PhenoFly Planning Tool [70].

### 3.4. High-Altitude Airborne and Satellite

The remote sensing community commonly uses specialised (expensive) high-quality imagers (usually line scanners rather than true imagers) mounted on aircraft flying at higher altitudes (often up to 2000 m)—this can allow simultaneous coverage of large trials (ideal for thermal studies), but at the expense of spatial resolution with few pixels wholly within any plot, so that edge effects of mixed pixels (where a pixel contains both plant canopy and background soil) become more serious.

Mixed pixels are particularly seriouswhen the size of the individual pixels are similar to or greater than that of the plant organs comprising the crop canopy [64, 71]. In the case of thermal imagery, mixed pixels typically result in the observed temperatures significantly biasing the background soil temperatures [71]. This edge effect can be very difficult to disentangle [72] in order to obtain the true crop signal.

Approaches to extract true canopy signal from digital imagery [71] are very scale dependent and range from whole pixel approaches, where image analysis is used to extract data only from crop pixels, to those methods such as unmixing and disaggregation that obtain information at a subpixel level. The expense and limited availability of such systems means that although they are useful for larger-scale agronomic studies, they are very unlikely to become the method of choice for breeders. Similarly, most readily available satellite imagery (e.g., MODIS of Landsat) has much too low a spatial resolution for phenotyping, while high-resolution imagery (WorldView, IKONOS, GeoEye, etc.) is no better than airborne, very expensive, and subject to limited availability due to scheduling problems or cloud cover [72].

## 4. Sensing Technologies

### 4.1. Reflectance, Multispectral, and Hyperspectral

Reflectance imagery for plant phenotyping ranges from conventional red/green/blue (RGB) images as used in consumer cameras (discussed in Section 4.2), through a range of sensors (often termed “Agricultural cameras”) with either one channel replaced by a near infrared (IR) reflectance channel [73] or with an additional IR channel, through multispectral sensors with a larger number of generally quite broad-band reflectance channels to true hyperspectral cameras that may have hundreds of precise narrow-band channels [72, 74]. The inclusion of an IR channel makes use of the high reflectance of plant material in the near IR and allows the calculation of a Normalised Difference Vegetation Index (NDVI) analogous to that originally derived for Landsat [75] or other vegetation indices (see [72], for a detailed discussion of options) as indicators of canopy density or leaf area index. In addition to light weight RGB cameras suitable for mounting on UAVs, a number of light weight multispectral “Agricultural cameras,” with more wavelengths, including IR, are now available for deployment on UAVs (e.g., Tetracam (http://www.tetracam.com), Parrot Sequoia (https://www.parrot.com), and MicaSense RedEdge https://micasense.com): some can be used with narrow wavebands chosen for specific purposes (e.g., Photochemical Reflectance Index or Flourescence studies of photosynthesis) (see Section 4.5). The use of multispectral imaging is a more powerful tool for crop phenotyping as it allows the selection of more appropriate spectral indices for any of a wider range of characters of interest [6]. The precision of estimates can often be improved with the use of the narrower spectral bands available with hyperspectral imagers. To date, the potential of multispectral and hyperspectral sensing has not been fully realised as they are commonly used in a relatively simple mode to derive only two band vegetation indices [72]. Nevertheless, the technology is potentially much more powerful, having been used to estimate photosynthesis using Solar-Induced Fluorescence (SIF) [76], Photochemical Reflectance Indices (PRI) [77], canopy senescence using time-resolved canopy spectral measurements [78], or indeed a range of photosynthetic traits (using a leaf-clip) [79] as well as other foliar traits across species, ecosystems, and forests [5, 80, 81]. It is worth adding an important word of caution here that the more complex the vegetation index used, the more critical is the need for good training data for the actual crop situation and the less likely that a standard vegetation index will be appropriate [82].

One example where substantial progress has been achieved is the use of hyperspectral sensing to phenotype maize for sensitivity to ozone damage [83], while there is also potential for monitoring soluble carbohydrates and other biochemicals [84]. As an alternative, there is evidence [85] that Fourier Transform Infrared (FTIR) spectroscopy (which extends further into the infrared) may be able to extract even more information on tissue biochemistry beyond what is possible with conventional hyperspectral sensing which only covers the range 400-1000 (-2500) nm. It is possible that radiation transfer modelling (e.g., using PROSAIL [86]) to account for varying radiation input, view and illumination angles, and leaf angle distribution could substantially improve the efficiency of extraction of key biochemical and physiological information, particularly with regard to canopy level measurements (as opposed to leaf-clip measurements where acquisition parameters are held relatively constant).

### 4.2. Red/Green/Blue (RGB) Imagery

RGB digital cameras are probably the primary tool for plant phenotyping imagery (especially from UAVs), because of their ready availability and low price, flexibility in application, and, for UAV operation, potentially low mass. Additionally, a major advantage of RGB imaging is the high resolution now available in consumer-grade cameras [87], and, rather obviously, that the visible spectrum enables humans to easily label features of interest and train object detection algorithms [88].

RGB cameras can be used in one of several modes, the simplest of which in terms of image analysis, and particularly suitable for lower resolution imagery such as from UAVs, is to take the average spectral signature (or colour) for all pixels deemed to be within the plot of interest. Thus far this is the most common approach to the use of RGB imagery, where it is usually used for the estimation of vegetation cover (usually as a proxy for leaf area index and hence use as a measure of crop growth), either through simple colour analysis to determine the proportion of vegetation and nonvegetation pixels [48, 89–94] or through the use of vegetation indices, especially when using Agricultural cameras. Even without the additional IR channel, RGB images are frequently enhanced for such studies by transformation of the colour space, for example, to enhance “greenness” [95]. Similarly, colour-space transformations such as Hue have been proposed for estimation of a range of characters that are expressed at a canopy level, such as chlorophyll content [96, 97] or leaf senescence [98], nitrogen (N) status [99], or disease susceptibility [100], all of which can be valuable for plant breeders [6].

Where higher-resolution imagery is available, for example, either from in-field buggies [46] or UAV, it becomes possible to undertake more sophisticated image analysis to identify individual plants [101, 102], leaves [103], tillers [104], or even wheat heads [105, 106]. This opens up a wide range of phenotyping possibilities that include selection for characters such as ear density and inflorescence size. Interestingly, stem diameter and stem density in wheat, as measured by RGB camera after grain harvest, were shown to be good predictors of head number and final biomass (when combined with biovolume measured before grain harvest) [107].

### 4.3. Photogrammetry and LiDAR

A further step that becomes possible with multiangular RGB imagery is to derive information on 3D canopy structure including characters such as leaf number and orientation, canopy height [108], or even crop volume as an indicator of biomass [109] using either conventional photogrammetry or “structure-from-motion” (SfM) analysis [110, 111], using dedicated software (e.g., Pix4D or Agisoft), or from other depth sensors such as LiDAR or time-of-flight cameras. Multiview imagery is incorporated in many laboratory imaging platforms [112, 113], allowing detailed 3D analysis of plant structure including leaf angle, size, rolling, and study of plant growth over time. Similar data can also be obtained using laser scanning or time-of-flight and light-field cameras. We do not intend to review these here.

Laser scanning or LiDAR (Light Detection And Ranging) is perhaps the most powerful current technique for detailed analysis of plant structure, especially when applied from more than one angle so occlusion is minimised. This is probably now the method of choice for proximal sensing from buggies [46, 48, 58] or tractors [57] for height and biomass surrogates. In the case of biomass in wheat, there is evidence of success at estimation of biomass from LiDAR, though it is notable that although reasonable correlations can be observed, inverse prediction from LiDAR readings is only weak and not robust across experiments [48, 57, 58].

The use of LiDAR and photogrammetry at close range (e.g., on buggies) enables the derivation of more subtle information that can be of interest to researchers, for example, leaf angle (distribution) (primarily obtained using LiDAR on buggies or photogrammetry in the laboratory). A key question is the detail of the information needed by breeders—how useful, for example, is detailed information on plant structure? Nevertheless, information on detailed canopy structure such as leaf angle distribution, while not being an objective in itself, may yet be useful for improving precision in the interpretation of simpler parameters such as canopy reflectance, because it allows the use of radiation transfer models such as PROSAIL [86] to enhance the data inversion for estimation of characters such as chlorophyll content, photosynthesis, or tissue biochemistry.

For the purposes of quantifying crop height and volume (as a biomass proxy), photogrammetry from UAV is arguably more suited to breeding-scale applications than LiDAR. This is due to the availability of turnkey consumer-grade UAVs equipped with RGB and often multispectral cameras, together with the availability of image mosaicking software and feature extraction algorithms. Additionally, as discussed herein and elsewhere [6, 114], it is possible to derive a wide range of quantitative information from RGB images. In contrast, LiDAR, although powerful, often requires engineering to capture, georeference, and process the data. Further, most LiDAR systems are mounted on a buggy or tractor, which may not have the required throughput for a breeding program. That said, a tractor-mounted LiDAR system enables the possibility of combining data collection with crop management activities [33, 57].

### 4.4. Thermal

Infrared thermography (IRT) can be a particularly powerful tool for studies of plant water relations and abiotic stress because canopy temperature (CT) is directly related to evaporation rate (which is itself dependent on stomatal conductance among other things) [115], so CT can be a powerful tool for screening for stomatal conductance. This relationship holds for any given environmental conditions, but in practice, it is essential to take account of environmental conditions at the time of imaging for any study involving canopy temperature (discussed further below).

**Figure 2:**
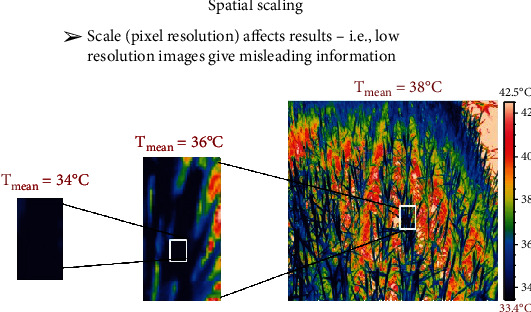
The importance of obtaining thermal data for just the leaves/canopy of interest is highlighted by the 4°C difference between the actual leaf temperature shown on the far-left and the resulting mean CT from the image on the far-right. The image on the far-right comprises all the pixels from the plot of interest and has a resulting mean CT of 38°C; the mean CT is clearly influenced by the soil pixels. The central image comprises considerably fewer pixels; however, the mean CT is 36°C and 2°C warmer than the image on the far-left that comprises only leaf pixels. The prevalence of incomplete ground cover impacting CT measures is more likely to be encountered in water-limited environments and in the planting configurations used in breeder's nurseries, owing to limited seed per genotype in early generations. Further work is required to develop routine methods for thresholding the background soil temperature and extracting thermal data exclusively from the plant material of interest. The thermal image was acquired using a FLIR® A655sc thermal infrared camera with a 13.1 mm lens.

**Figure 3:**
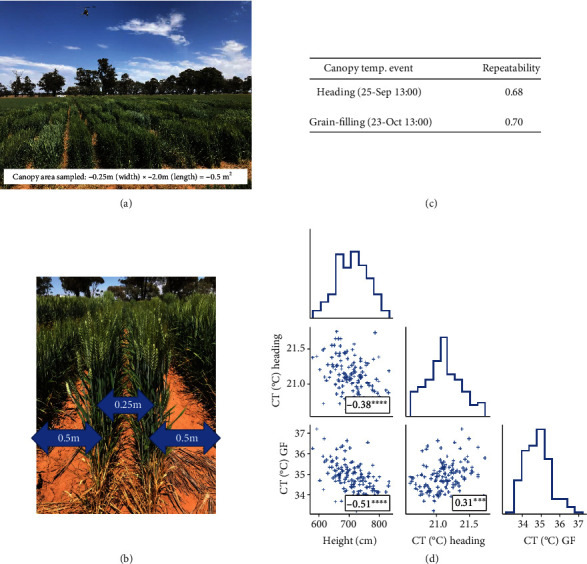
Example application of airborne thermal imagery to quantify canopy temperature (CT) on small plots using the previously described airborne thermography method [64]. (a) The experiment, comprising two replications of 133 commercially released wheat varieties, and manned-helicopter used to capture CT. (b) Each plot comprised two rows, 0.25 m apart, about 2.7 m in length and with 0.5 m interplot spacing. This plot configuration is utilised by an Australian breeding company and is therefore representative of the small plots commonly used in breeding nurseries to evaluate early-generation germplasm. Results are shown for (c) two CT events made during the heading and grain-filling growth stages as repeatability estimates and (d) Pearson correlations between genotype BLUPs for the two CT events and canopy height. Given the smaller plot size, the (c) high repeatability estimates for the two events are encouraging as the thermal camera resolution (640 × 480) can limit the CT data quality. Note the significant association between (d) CT events and the high negative associations between CT and height, despite only a small range in height of about 0.2 m. Therefore, to better relate CT to stomatal conductance, further research is required to develop methods that can account for variation in height when sampling CT, eliminating boundary-layer effects. Thermal images were acquired using a FLIR® A655sc thermal infrared camera, with a 13.1 mm lens, mounted on a manned-helicopter flying at an altitude of 50 m, resulting in a pixel size of 6.5 cm. Approximately, 90 to 110 pixels were acquired per plot by selecting an inner section of the plot canopy: three or four pixels across the width of the plot (*ca.* 0.25 m) and *ca.* 30 pixels along the length of the plot (*ca.* 2.0 m). Repeatability and genotype BLUPs were estimated at each individual sampling time using the SpATS package [160].

There are many published examples where handheld thermal sensing has been used in phenotyping for stomatal conductance or drought adaptation (e.g., [116–120]). The enhanced capacity and much greater heritabilities provided by the use of imaging approaches, especially from airborne platforms, mean that this approach now shows real promise for high-throughput phenotyping of water productivity using manned [43, 64, 121] or unmanned aircraft [122]. There are, however, many critical aspects to using this technology successfully. For example, to obtain true canopy temperatures, it is necessary to correct for errors relating to mixed pixels [71], particularly from incomplete ground cover (Figure 2). This issue becomes more pertinent when assessing genotype performance under water limitation, where greater incomplete ground cover is more likely, than environments with high water supply. Similarly, because of the sensitivity of CT to environmental conditions (for a thorough analysis, see [123]), it is necessary to devise effective methods for normalisation of data for environmental variation [37] or else for all images to be obtained as rapidly as is possible with airborne sensors which can greatly reduce the time taken to complete imagery of a field. Additionally, the prevailing environmental conditions, particularly vapor pressure deficit (VPD) and solar radiation, can influence CT repeatability estimates, with higher repeatability more likely with higher VPD and solar radiation [43]. The higher CT repeatability observed under such conditions, which are more likely to occur in the afternoon and during the grain-filling period, is possibly (but not necessarily) because differences in stomatal conductance between genotypes were more pronounced. The latter is plausible when considering energy balance theory [72], whereby for a given stomatal conductance, CT is linearly related to VPD and solar radiation. Therefore, any differences in stomatal conductance are likely to result in larger differences in CT with greater VPD and solar radiation. A further complication that needs to be considered is that canopy temperature depends not only on stomatal conductance but also on canopy height [16, 119, 120, 124, 125], and morphology such as the presence and form of ears also affects results. In the case of height, negative associations with CT (where taller canopies are cooler) are typically attributed to the greater coupling between canopy and atmosphere [126] for taller genotypes. It is important to note that even small differences in height can influence CT, as per the example shown in Figure 3 where despite only a 0.2 m range in height, there were significant associations between CT and height. Thus, although there is good evidence that in general taller canopies have lower canopy temperatures [16, 119, 120, 124, 125], the inclusion of major height genes (as factors) together with quantitative variation in plant height as covariates substantially improved the genetic correlation between CT and grain yield in populations varying for plant height [120]. Notably, in some populations, similar quantitative trait loci (QTL) for height and stomatal conductance have been reported, where genotypes with *Rht* dwarfing genes (controlling plant height) had higher conductance in spite of their shorter stature and higher canopy temperatures [120]. This indicates that selection for canopy height cannot be used as an indirect indicator of stomatal conductance. Therefore, for the effective deployment of CT within plant breeding, further work is required to accommodate height variation when sampling CT.

There is an important trade-off between acquisition height and pixel size (sometimes called ground resolution) with thermal cameras, particularly when mounted on airborne platforms. This trade-off, while still applicable to any imaging sensor, is typically more pronounced with thermal cameras which are generally limited to a pixel resolution of 640 by 480 (although higher-resolution cameras are available, they are considerably more expensive). For example, the pixel size of a FLIR®A655sc thermal camera with a 13.1 mm (45°) lens is about 7 cm at 60 m height and about 15 cm at 120 m (https://flir.custhelp.com). Lower heights or a narrower-angle lens would be required to obtain leaf-sized pixels for cereal crops. There is a similar trade-off with speed of platform movement that depends on the effective shutter speed (integration time) of the camera [71]. As mentioned earlier, in the case of thermal cameras, greater resolution is currently possible but considerably more expensive. For example, the FLIR®X8500sc SLS (https://www.flir.com/products/x8500sc-sls-lwir/) is a cooled thermal camera with a spatial resolution of 1280 × 1024, a thermal resolution (pixel sensitivity) of 0.04°C, and minimum integration time of 270 ns (however, the FLIR®X8500sc SLS is approximately an order of magnitude more expensive than the FLIR®A655sc uncooled thermal camera). Nevertheless, such a camera offers potential for high-quality CT measurements on the small planting configurations used in breeder's nurseries that can comprise single or multiple rows or even single plants (Figure 1). However, it is important to recognize that the physical size of the respective space-plant (e.g., single plants, short-single or short-double rows, and microplots) has both technological effects on the screening process (e.g., problems related to pixel size and impacts on crop boundary layer in the case of CT), as well as impacts on the expression of some crop performance-related characters (discussed elsewhere [25, 26, 30, 127]). That said, the example in Figure 3 demonstrates the successful application of airborne thermal imagery using the FLIR®A655sc thermal camera on small two-row plots (0.25 m by 2.0 m) with the same configuration as that used by an Australian breeding company to evaluate their early-generation germplasm; note the high repeatabilities and reasonable association between the CT events.

Canopy temperature measurements can be used experimentally, in a relative mode which is particularly suitable for most phenotyping applications [49], or it can be used to provide information on absolute evaporation or stomatal conductance [128]. Unfortunately, it is still unclear whether, at a given growth stage, a high canopy temperature (indicating water conservation) or a low temperature (indicating continued stomatal opening) is going to be optimal for drought performance in any particular environment. Nevertheless, with the exception of extremely water-limited environments, at least one study in wheat has shown that selection for yield in irrigated and low-stress environments can confer performance across to more water-limited environments [129]. Therefore, indicating that selection for cooler CT (greater conductance) in irrigated nurseries, during the grain-filling period when repeatability is generally higher [43], may translate across through improved yield in less favourable environments. In addition, several research groups working across multiple crops have reported variation in the capacity of genotypes to limit transpiration rate (TR) in response to increasing VPD [130–134]. This trait, referred to herein as limited-TR, is a water-saving trait that essentially reduces transpiration when VPD exceeds a threshold, so that water is conserved until later in the season. However, limited-TR is commonly evaluated under precisely managed experimental conditions, in a glasshouse or growth cabinet with controlled temperature and VPD, with TR determined by weight with the soil covered and normalised by leaf area. That said, promising results were obtained using thermal imagery to evaluate limited-TR across 12 cowpea genotypes [131]. In summary, although direct estimates of TR from CT [42] to evaluate limited-TR at the scale of a breeding program are plausible, the opportunity of using CT to assist selection for performance under water limitation requires more testing.

### 4.5. Other Technologies

A range of other technologies are being developed for phenotyping, though some are more suitable for laboratory/controlled environment situations, especially where plants are moved on robotic systems to a sensing station. These include the use of X-ray or Gamma-probes for biomass or the use of NMR or MRI for studies of sap flow or root anatomy [135], though at present the latter approaches are low throughput. Multispectral fluorescence imaging appears to hold promise for stress monitoring for phenotyping [136–139], though the relationships found tend to be empirically rather than mechanistically based, so are not particularly robust to environmental or growth conditions. Similarly, chlorophyll fluorescence has great potential for phenotyping of photosynthesis, but the more precise modulated measurements are only suited for the laboratory or close range field systems because the inverse square law limits operating distances [82]. The development of Laser-Induced Fluorescence Transient (LIFT) technology, however, can potentially allow fluorescence photosynthesis measurements to be made at distances of several meters [140], for example, from buggies. An alternative, though less precise approach in the field is to use SIF (see above) based on narrow-band hyperspectral sensors from airborne platforms [141].

## 5. Data Handling

Data handling is arguably a major limiting factor to making truly “user-friendly” phenotyping systems. At the core of phenomics data handling is the need to convert raw sensor data into biologically relevant metrics and georeference the data to individual experimental units within a given experimental design. Therefore, a critical feature of phenomics is the development of software that enables easy transfer of data and metadata between different phenotyping platforms and the introduction of standardized reporting to facilitate archiving and analysis of the enormous amounts of data collected [142]. Example applications of such systems include, but are certainly not limited to, thermal imagery [64], LiDAR [48], high-frequency time-series data [143], and RGB from UAV [144]. However, the development of data handling systems that can reliably georeference measurements to individual plants, short-single, or short-double rows (as used in breeder's nurseries) (Figure 1) is particularly important to enable the reliable deployment of phenomics into large-scale breeding programs. Arguably, for phenomics applications in plant breeding, the operation of the system needs to be completely automatic with minimal user inputs required at any stage. Clearly, data handling will continue to be an important area for applied phenomics researchers, as the recent advances in imaging have rather outstripped the ability both to manage and archive data and the necessary metadata and importantly to automatically analyze the results to provide outputs directly that are useful to breeders and researchers. Therefore, the development of effective data management systems and “user-friendly” analysis tools is an important enabling technology that needs application of intensive resources. Also, of prominent importance is the statistical modelling required to ultimately predict genotype performance for a particular target environment. This topic, although beyond the scope of this article, was recently reviewed [145].

## 6. Field Phenomics Application to Breeding

Although the range of variables that can be targeted by phenotyping systems is enormous, there is a critical need for phenotyping efforts to target breeders' needs; there is a widespread perception that much of current research is more for physiologists and prebreeders than for breeders. Furthermore, it seems that much of the work is still driven by available technology rather than by breeders' (or even physiologists') requirements. This is a common part of the problem for service systems or enabling technologies which are liable to fail to satisfy or keep up with the user requirements unless great care is taken, especially if the service is remote from the end users. It is particularly important therefore that phenomics researchers are attuned to breeder and prebreeder requirements through appropriate mechanisms—possibly by surveys, interviews, and regular face-to-face interactions and ideally through jointly developed research projects. It will remain critical to continually assess the purpose of phenomics research and its relevance to crop improvement. For example, breeders and prebreeders may be less interested in some of the more detailed information that can be obtained from image analysis (e.g., relating to leaf number and orientation) than in some of the relatively simpler outputs such as surrogates for LAI or photosynthesis or transpiration rates. Indeed it is encouraging that the application of field phenomics (CT and spectral indices) has improved the accuracy of pedigree and/or genomic selection models in several studies [36, 121, 146, 147]. Further, a study in wheat reported promising results relating spectral indices measured on a range of space-plant configurations (one to three rows per plot) to grain yield [148]. Additionally, encouraging results were reported [35] from indirect selection for sugar cane yield based on remotely sensed canopy height, canopy cover, and NDVI. Nevertheless, despite promising results, the application of field phenomics in plant breeding to assist decision-making is not commonplace and further work is therefore required that is targeted at plant breeding applications. Practical considerations are important, including the development of inexpensive tools that can quickly quantify many individual genotypes across multiple locations [6]. Furthermore, it is worth evaluating the potential benefit, if any, of automation for variables that are easily scored visually by eye. Examples include flowering time, height, and canopy architecture (erectophile versus planophile). Although these traits are typically assessed manually, by breeders walking their trials, augmenting this process with handheld smartphone apps may improve effectiveness. However, it may be that remote and proximal sensing is of greater benefit for variables that cannot be seen easily or those that are highly subjective to estimate by eye. Thus, additional work is required to assess the benefits and trade-offs of specific approaches, for example, assessing the cost of measuring multiple traits with the same platform (e.g., a drone carrying an RGB sensor) versus the labour saved and realised genetic gain. Such propositions are amenable to economic assessment, similar to that presented by Brennan and colleagues [149]. The latter study demonstrated a high likelihood of economic benefit to the CIMMYT breeding program through indirect selection for yield based on screening for stomatal aperture-related traits. Relatedly, Awada et al. developed an economic model to analyze breeder's decision-making regarding the adoption of phenomics technology [32]. Their model highlights an obvious but salient point for phenomics researchers: adoption requires that the return from adopting phenomics is greater than the cost of phenomics and the profit from the existing system. Testing such an adoption model, which is theoretical at this stage, with real-world data is a complex undertaking requiring domain expertise in behavioural psychology and social sciences, but potentially invaluable for understanding the requirements for phenomics adoption by breeders and guiding research effort in phenomics.

As major yield determinants, susceptibility to pests and diseases is a key area where good phenotyping techniques are required [150], but much work is still needed before effective automated screening can be performed [151]. Disease diagnosis is likely to require detailed analysis of patterns of colour on individual leaves, which is feasible at a laboratory scale using image analysis approaches [152, 153]. At the field scale, quantitative resistance to *Septoria tritici* blotch in winter wheat populations was evaluated by a rather manual, but effective, process: infected leaves were sampled and then scanned on a flatbed scanner, and the images were later analyzed for lesion characteristics [154–156]. As such, the current approaches generally involve a two-stage segmentation [157]. Firstly, the leaves of interest are segmented from the background, for example, by manual image processing [157] or, in the aforementioned examples [154–156], by providing a uniform background to enable automatic segmentation. Secondly, the disease symptoms are separated from the healthy tissue as a means of identifying different diseases from the pattern of lesion and for scoring the severity of infection. For this application, the sensing is often based on the pattern of variation detected on the leaf, where conceivably, the use of smartphones and artificial intelligence may alleviate the challenge of disease identification and scoring. This type of inference is in contrast to sensing based on an average spectra derived from a leaf/plant or plot, where disease-specific spectral indices have been developed [151] or where time-resolved canopy spectral reflectance measurements are used to determine the presence and severity of leaf foliar diseases [158]. The latter approach required ground-truth measurements of disease presence and severity at frequent time intervals to effectively distinguish between the spectral signal associated with the foliar disease from that associated with canopy development. The outlook for improved disease phenotyping at the scale required for plant breeding is promising given the increased availability of UAVs equipped with high-resolution cameras and advanced feature extraction algorithms. To this end, promising results at the plot scale were recently obtained using feature extraction from hyperspectral imagery, captured from a UAV, for detecting diagnostic patterns related to disease severity of yellow rust in wheat [159].

## 7. Conclusions

The application of phenomics in plant breeding to assist with selection of genotypes is seen as an important step for ongoing crop improvement. However, this application requires concerted effort on the part of researchers to adapt and develop phenomics methods to support breeding programs. In particular, key considerations for phenomics researchers are as follows: how related the phenotype is to the breeding target of a particular breeding program, whether the phenotype is sufficiently repeatable, at a given sampling time, and heritable across years and locations; how amenable a particular method is to screening large numbers of individuals in breeder's nurseries at an acceptable cost and across multiple locations; and the level of data handling required to derive biologically meaningful information for each individual genotype. Finally, realising the full benefits of phenomics to support decision-making within breeding programs will clearly depend on additional enabling technologies. Not least of which are crop and economic optimisation models to strategically guide the deployment of phenomics within a particular plant breeding program.
